# 2D-QSAR study of some 2,5-diaminobenzophenone farnesyltransferase inhibitors by different chemometric methods

**DOI:** 10.17179/excli2015-177

**Published:** 2015-03-30

**Authors:** Saeed Ghanbarzadeh, Saeed Ghasemi, Ali Shayanfar, Heshmatollah Ebrahimi-Najafabadi

**Affiliations:** 1 Drug Applied Research center and Faculty of Pharmacy, Tabriz University of Medical Sciences, Tabriz, Iran; 2 Department of Medicinal Chemistry, School of Pharmacy, Guilan University of Medical Sciences, Rasht, Iran; 3 Department of Medicinal Chemistry, Faculty of Pharmacy, Tabriz University of Medical Sciences, Tabriz, Iran

**Keywords:** QSAR, multiple linear regression, artificial neural network, support vector machine

## Abstract

Quantitative structure activity relationship (QSAR) models can be used to predict the activity of new drug candidates in early stages of drug discovery. In the present study, the information of the ninety two 2,5-diaminobenzophenone-containing farnesyltranaferase inhibitors (FTIs) were taken from the literature. Subsequently, the structures of the molecules were optimized using Hyperchem software and molecular descriptors were obtained using Dragon software. The most suitable descriptors were selected using genetic algorithms-partial least squares and stepwise regression, where exhibited that the volume, shape and polarity of the FTIs are important for their activities. The two-dimensional QSAR models (2D-QSAR) were obtained using both linear methods (multiple linear regression) and non-linear methods (artificial neural networks and support vector machines). The proposed QSAR models were validated using internal validation method. The results showed that the proposed 2D-QSAR models were valid and they can be used for prediction of the activities of the 2,5-diaminobenzophenone-containing FTIs. In conclusion, the 2D-QSAR models (both linear and non-linear) showed good prediction capability and the non-linear models were exhibited more accuracy than the linear models.

## Introduction

Malaria is a deadly disease which is cause of more than 2-3 million deaths every year in the world and is estimated to be endemic in over 100 different countries. There are over 200 different *Plasmodium* species, but only 4 known types actually cause human malaria. *Plasmodium falciparum *is more dangerous and deadly than other species of *Plasmodium*species that can cause malaria in human (Eastman et al., 2007[9]; Olepu et al., 2008[26]; Xie et al., 2006[35]).

Because of problems with available drugs (Chloroquine), such as drug resistance, finding new drugs with new mechanisms for treatment of malaria is required (Gupta and Prabhakar, 2008[17]; Xie et al., 2006[35]).

The RAS proteins belong to a family of related polypeptides that are present in all eukaryotic organisms from yeast to human. The RAS proteins are critical in signal transduction pathway and in cell growth. Several studies on RAS proteins have showed that some post-translational modifications are essential for its biological activity (Ghasemi et al., 2013[14]; Lu et al., 2007[24]; Puntambekar et al., 2008[27]). The first step of these modifications is farnesylation by farnesyltransferase enzyme (FTase). FTase is a heterodimeric metalloenzyme that contain a zinc ion (Gilleron et al., 2007[15]; Puntambekar et al., 2008[27]; Xie et al., 2006[35]). FTase adds a C-15 farnesyl group from farnesyl pyrophosphate (FPP) to the cysteine of the CAAX sequence (C=cys, A=an aliphatic amino acid, X is typically Met) in the carboxyl terminal of RAS proteins (Bolchi et al., 2007[4]; Equbal et al., 2008[10]; S Ghasemi et al., 2013[13][14]; Lu et al., 2007[24]; Tanaka et al., 2007[31]).

It has been showed that farnesyltranaferase inhibitors (FTIs) can inhibit the growth of *Plasmodium falciparum *in human red blood cells (Ohkanda et al., 2001[25]). Therefore, these compounds can be used as antimalarial agents against *Plasmodium falciparum *(Shayanfar et al., 2013[29]).

Several classes of antimalarial FTIs have been synthesized such as 2,5-diaminobenzophenone derivatives, biphenyl derivatives, tetrahydroquinoline and etc. (Ohkanda et al., 2001[25]; S Olepu et al., 2008[26]).

The drug development contributes to high cost and long time. Quantitative structure-activity relationship (QSAR) approach as a computational methods can be used to predict drug biological activity by finding a correlation between the structures and the activities of drugs, and therefore decreases the cost and time of the drug development (Shayanfar et al., 2013[29]; Yee and Wei, 2012[36]). This methods are based on correlation between molecular properties and differences in the features of the molecules (Jain et al., 2012[19]).

Two-dimensional (2D) and three-dimensional (3D)-QSAR are the most common QSAR models. 2D-QSAR models investigate correlation between the activities of active molecules and structures without regarding the three-dimensional conformations of the molecules. However, 3D-QSAR models consider the 3D conformations of the molecules (Shayanfar et al., 2013[29]). 

Several studies by 2D-QSAR modeling were performed for prediction of FTIs biological activities. Freitas and Castilho (2008[11]) investigated the activities of tetrahydroquinoline FTIs using multiple linear regression (MLR) models. Gupta and his coworker also correlated FTI activities to tetrahydroquinoline analogues structures with 2D-QSAR model with the Combinatorial Protocol in Multiple Linear Regression (CP-MLR), a filter based variable selection procedure (Gupta and Prabhakar, 2008[17]). Modeling studies were performed for some thiol and non-thiolpeptidomimetic inhibitors using artificial neural networks (ANN) and radial distribution function (RDF) approaches by Gonzalez et al. (2006[16]). Recently Gaurav et al. (2011[12]) and Shayanfar et al. (2013[29]) also studied QSAR of imidazole containing FTIs.

Despite of the many benefits of 3D-QSAR models, 2D-QSAR models have some beneficial advantages. In 2D-QSAR models it is not necessary to align the structures that can create some limitation in 3D-QSAR. Furthermore, development of 2D-QSAR models is very faster and easier than 3D-QSAR models (Shayanfar et al., 2013[29]).

Literature review indicated that, no 2D-QSAR study has been reported for 2,5-diaminobenzophenone-containing FTIs. Therefore in the present work, 92 FTIs with 2,5-diaminobenzophenone scaffold were used to develop 2D-QSAR models by various chemometric methods. Multiple linear regression (MLR), ANN and support vector machine (SVM) methods were used to predict the IC_50_ of the 2,5-diaminobenzophenone-containing FTIs. Genetic algorithms-partial least squares (GA-PLS) and stepwise-regression methods were used to select molecular descriptors. Internal validation method was used for confirmation of the validities of the developed models. 

## Material and Methods

### Data Set

**Figure 1:**
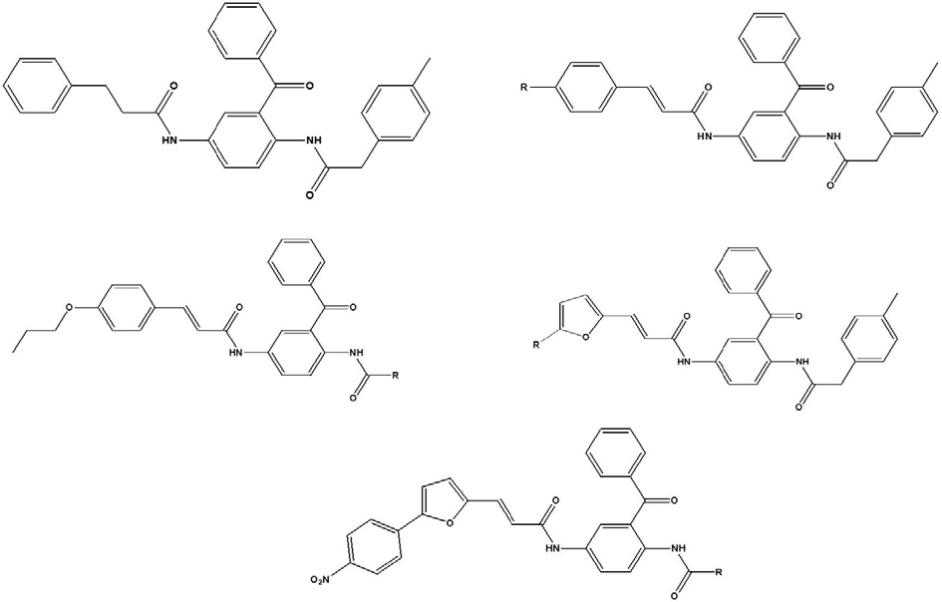
Structures of the studied 2,5-diaminobenzophenone-containing farnesyltranaferase inhibitors

The pIC_50_, negative logarithm of the IC_50_ (half maximal enzyme inhibitory concentration), values of the ninety two 2,5-diaminobenzophenone-containing FTIs were collected from the literature (Xie et al., 2006[35]). This data set is formed of the five different groups of 2,5-diaminobenzophenone-containing FTIs. Chemical structures of these compounds are shown in Figure 1(Fig. 1). In order to compare the results of the present study (2D-QSAR) with previous 3D-QSAR study, the same carefully-selected training and test sets were employed in the model development (Xie et al., 2006[35]).

### Molecular descriptors

The structures of the all studied compounds were drawn using Hyperchem 8.0 software and pre-optimized with the molecular mechanics force field (MM+) method to calculate molecular descriptors. Subsequently, AM1 semiempirical calculations were performed for optimization of the 3D geometries of the molecules with the Polak-Ribière (conjugate gradient) algorithm.

Finally, Hyperchem 8.0 software was fed into the Dragon 3.0 software and the molecular descriptors of these compounds were calculated.

### Descriptors selection

With the aim of reduction in the number of descriptors, the descriptors belonging to 74 compounds in the training set with higher than 50 % repeated values or collinear descriptors (*R *> 0.9) were excluded and further reduction in the number of descriptors was performed with Genetic algorithm and partial least square, a valuable tool for data reduction, (GA-PLS). GA simulates the process of natural evolution and has been used commonly as an acceptable method for reducing the number of descriptors (Dastmalchi et al., 2008[7]; Habibi-Yangjeh, 2009[18]; Soltani et al., 2010[30]). The MATLAB 7.8 software was used to run the GA-PLS method developed by Leardi et al. (2002[22]). Population size is one of the major factors which affect the performance of the algorithm and it is necessary to have good population to produce optimal result in quick time. The population size of genetic algorithms in this study was considered as 100. Ten percent of the descriptors with top scores were selected and the descriptor selection was performed using stepwise regression. High correlations with response and low inter-correlation between descriptors (using Pearson correlation) were considered as selection criteria before stepwise regression.

## Model Building

### MLR Model

The selected descriptors were employed to develop a MLR equation using SPSS 16 software. Statistical properties of the proposed equation including correlation coefficient (R), adjusted correlation coefficient (Radj), standard error of estimate (SEE), probability values (p-value) of each descriptor, and Fischer statistic or variance ratio (F), recommended by Dearden et al., (2009[8]) were obtained. The proposed model was validated using the leave one-out (LOO) method to evaluate prediction capability of the model.

### ANN with the Levenberg-Marquardt Algorithm

ANN, which mimics human brain process information, is useful in detecting complex non-linear relationship between a set of inputs and outputs. Briefly, the general structure of ANN has one input layer, one or more hidden layers and one output layer. Each layer has some units corresponding to neurons. The units in neighboring layers are fully interconnected with links corresponding to synapses. The strengths of connections between two units are called 'weights'. Selected descriptors are neurons of the input layer, pIC_50_ values of compounds are the output neurons and a three layer networks with three neurons in the hidden layer was designed. ANN learns an approximate non-linear relationship by a training procedure, which involves varying weight values. Training means a search process for the optimized set of weight values, which can minimize the squared error between the estimated and experimental data of units in the output layer. The number of training cycles was selected on the basis of the Mean Squared Error (MSE) of the validation subset, which prevents the network from over-training (Jalali-Heravi et al., 2008[20]). Neural networks for modeling in conjunction with genetic algorithms have proved very powerful for optimization. There are different algorithms for weight update functions in the literature. In the recent QSAR studies, the Levenberg-Marquardt algorithm was considered as one of the most effective algorithm (Arab Chamjangali, 2009[1], 2007[2]; Jalali-Heravi et al., 2008[20]) . In this study, we used the nftool (network-fitting tool) toolbox of MATLAB 7.8 software for training of the network. This toolbox is user-friendly and uses Levenberg-Marquardt back propagation algorithms (Trainlim) for ANN training. For training a valid network and preventing over fitting the 56 data points of the training set, described for MLR, were randomly classified into training (70 %), validation (15 %) and test (15 %) sets. 

### Support Vector Machine 

SVM is a new and very promising classification and regression method developed by Vapnik (2000[34]) SVM have been successfully used to solve classification and correlation problems, such as cancer diagnosis, identification of HIV protease cleavage sites, protein class prediction, etc. SVMs have also been applied in chemistry and QSAR studies (Cheng et al., 2010[5]; Darnag et al., 2010[6]; Shahlaei et al., 2010[28]; Vapnik, 2000[34]). In this method a hyperplane is constructed in a multidimensional space which provides the minimum error by employing a non-linear kernel function for classification or regression tasks. Some parameters should be optimized in SVM analysis include the capacity parameter (C) that is a regularization parameter that adjusts maximizing the distance from the hyperplane to any training set data points and minimizing the error. ε is another parameter which is related to noise in the data. A common type of kernel function is a radial basis function (RBF) (Asadpour-Zeynali and Soheili-Azad, 2010[3]; Katritzky et al., 2010[21]; Louis et al., 2010[23]; van de Waterbeemd and Testa, 2008[33]). This function has a parameter (γ) which should be optimized and controls the generalization ability of the SVM. The C and ε parameters were optimized using the leave-many-out cross-validation method. SVM was performed using STATISTICA 7 software.

## Results and Discussion

### Selection of descriptors

The details of the selected descriptors by using GA-PLS and stepwise regression (where less than 1 descriptor per 9 compounds was selected) are shown in Table 1(Tab. 1). Results indicated that a mixture of 2D and 3D descriptors showed the best predictability for the pIC_50_ value of the studied structures. Three of the used descriptors are topological descriptors, which is a connectivity index is a type of a molecular descriptor that is calculated based on the molecular graph of a chemical compound. In addition, BCUT as another 2D descriptors are selected (Todeschini and V Consonni, 2008[32]). On the other hand, four 3D descriptors including RDF, WHIM and GETAWAY were in the selected descriptors. According to the selected descriptors, it was found that both of the volume, shape and polarity of the molecules were important for the activity of the studied compounds. 

A correlation matrix indicated that there was no intercorrelation (R < 0.6) between the selected descriptors (Table 2(Tab. 2)) which showed that the selected descriptors were linearly independent and as a result could be used simultaneously in the QSAR models development.

### Model building using different methods

**Figure 2:**
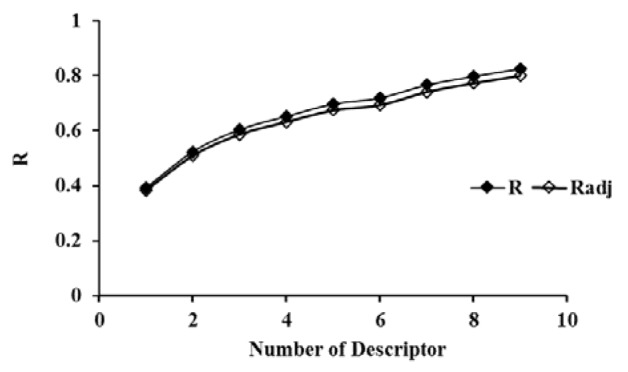
Effects of the number of descriptors on R and Radj values.

The selected descriptors were used for the QSAR models development by using MLR, ANN as well as SVM. Based on the obtained results, a linear model, as the simplest and most straightforward model was proposed. The standard error of estimate, coefficient and the p-value of the selected descriptors of the most accurate MLR model were presented in Table 3(Tab. 3). Furthermore, statistical information which is necessary to validate QSAR models are presented in Table 4(Tab. 4) for the proposed models in the present study. The results indicated that there was no significant difference between Rand Radj and the correlation coefficient was acceptable (P < 0.05)*.*The influence of the number of descriptors on Rand Radj for the developed model are presented in Figure 2(Fig. 2). The increase in the descriptor number resulted in increase in Radj value confirmed the influence of all the selected descriptors (Dearden et al., 2009[8])

The obtained data was used to develop an ANN model with three optimal hidden neurons. Table 4(Tab. 4) shows the statistical parameters of the developed ANN model for the data set including training, validation and test sets. There are no significant changes between statistical properties of these sets.

Selected descriptors were used to develop SVM models. The STATISTICA 7 software was employed for optimization of the SVM parameters (C, ε and γ) with 10-fold cross-validation. A robust model can develop by selecting parameters which give the lowest error. The optimized values of C, ε and γ were 7, 0.001 and 0.1, respectively. The statistical properties of the proposed SVM model for the training set are listed in Table 4(Tab. 4). 

Experimental and predicted pIC_50_ as well as absolute error values using MLR, ANN and SVM models are summarized in Table 5(Tab. 5).

**Figure 3:**
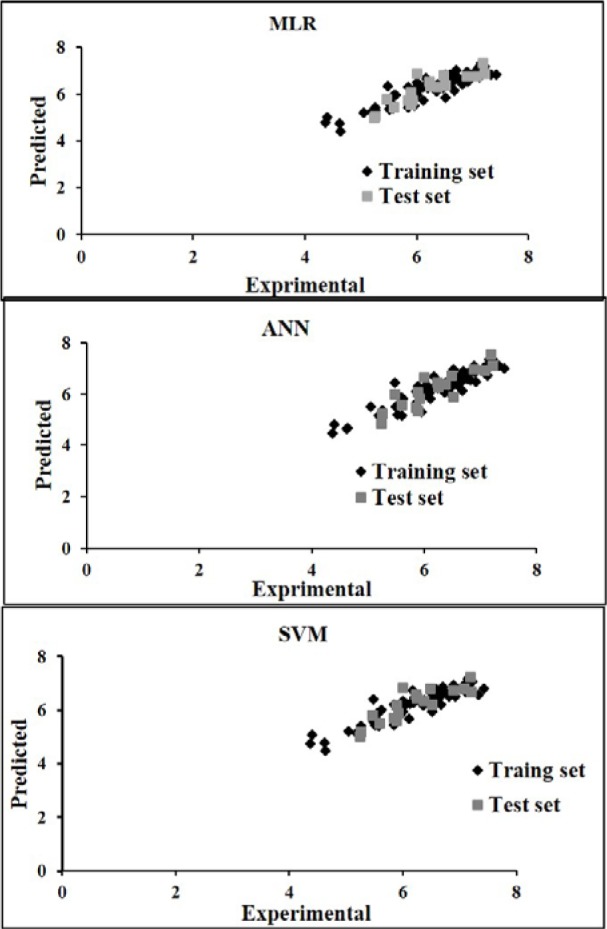
Experimental versus predicted pIC_50_ values using MLR, ANN and SVM models

Figure 3(Fig. 3) shows the experimental versus predicted values for training (74 data points) and test sets (18 data points) using MLR, ANN and SVM models. The AAE values of training and test compounds are listed in Table 6(Tab. 6). These data indicated that the developed models have good predictability. The AAE's of the ANN and SVM models were better than those of the MLR model and accordingly, as shown in Tables 4(Tab. 4), R values of the ANN and SVM models (as non-linear models) were also greater than that of the MLR model (as linear model) indicating that the SVM and ANN models are more accurate than the MLR model.

## Conclusion

Different chemometric methods were used to developed QSAR models to predict the activities of 2,5-diaminobenzophe-containing FTIs employing a collection of 2D and 3D descriptors to display the FTI structures. The obtained results demonstrated that the volume, shape and polarity are important parameters for the activity of the studied compounds. Furthermore, developed 2D-QSARmodels by using linear (MLR) and especially nonlinear(ANN and SVM) methods can be used to predict the activities of FTIs with high accuracy. In conclusion, the proposed models could be used in drug design to evaluate novel 2,5-diaminobenzophe-containing FTIs.

## Conflict of interest

The authors declare that they have no conflict of interest.

## Figures and Tables

**Table 1:**
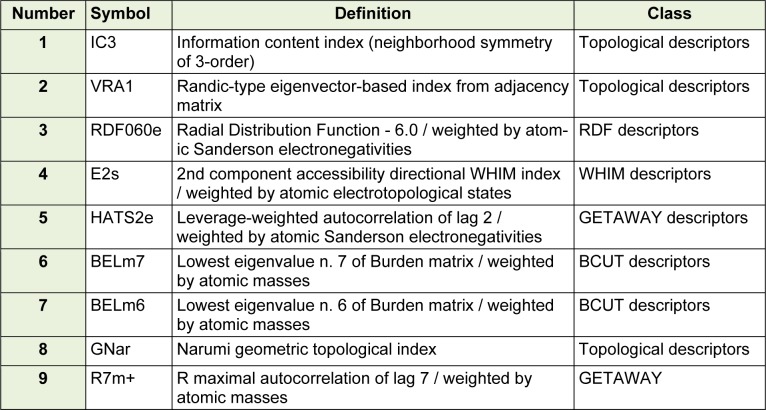
Selected descriptors by GA-PLS and stepwise regression from DRAGON software

**Table 2:**
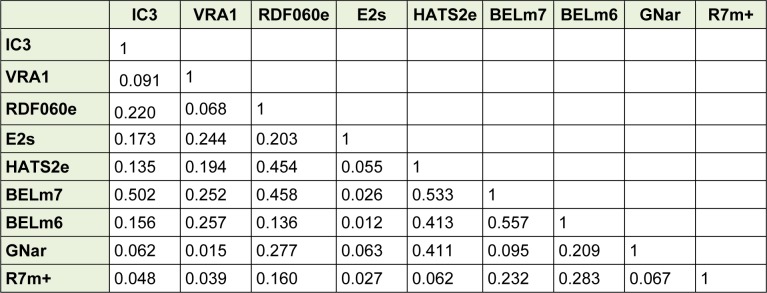
Correlation matrix between selected descriptors

**Table 3:**
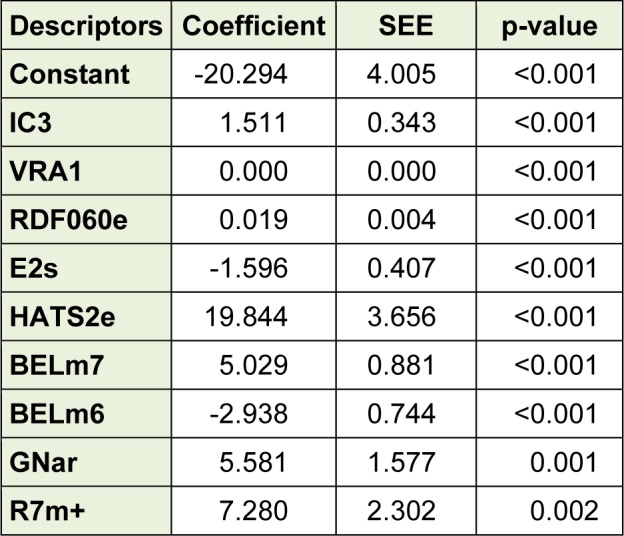
Coefficients and standard error of estimate and the p-value of the selected descriptors of the most accurate MLR model

**Table 4:**
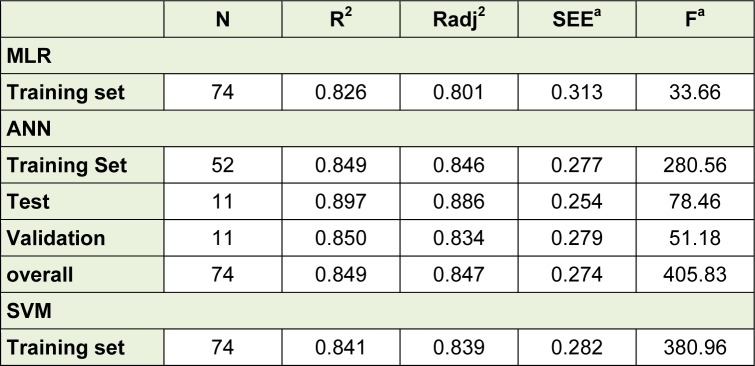
Statistical information for the proposed models for the Training Set

**Table 5:**
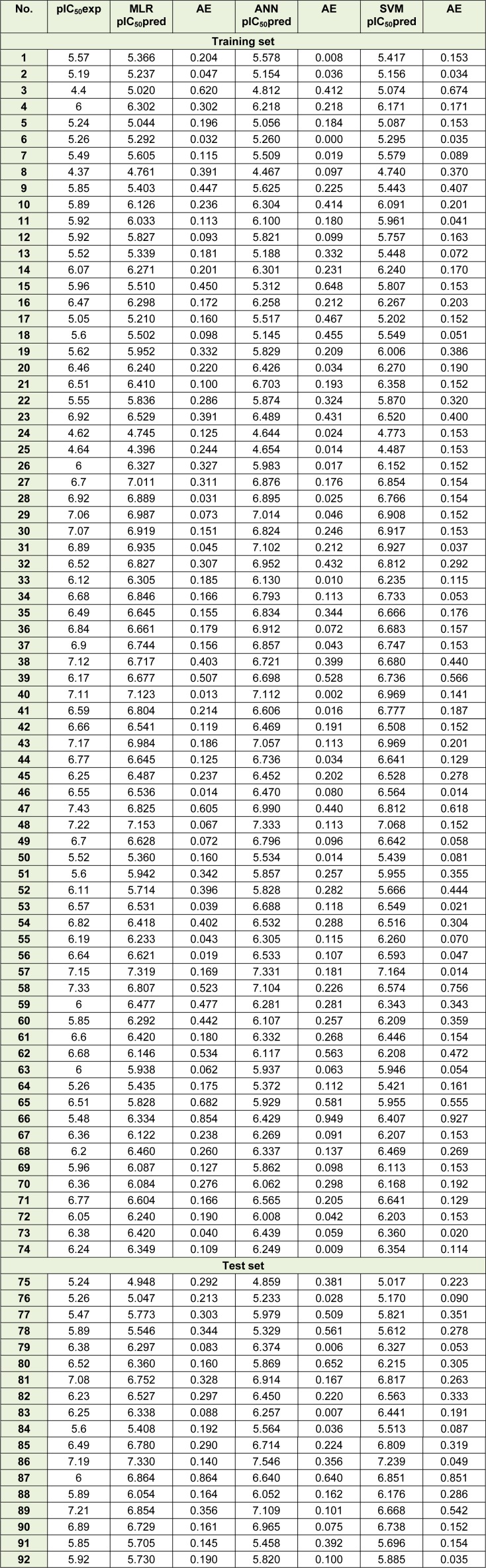
Experimental (exp) pIC_50_, predicted (pred) IC_50_ and absolute error (AE) values of 74 training and 18 test set compounds

**Table 6:**
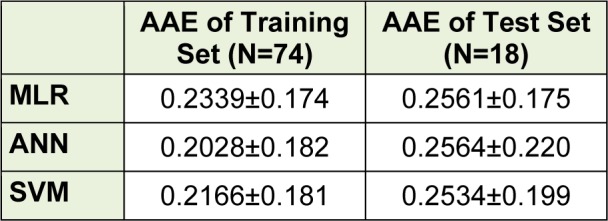
AAE's of the proposed models using different chemometrics methods

## References

[1] Arab Chamjangali M (2009). Modelling of cytotoxicity data (CC50) of anti-HIV 1-(5-chlorophenyl) sulfonyl)-1H-pyrrole derivatives using calculated molecular descriptors and Levenberg–Marquardt Artifical Neural Network. Chem Biol Drug Des.

[2] Arab Chamjangali M, Beglari M, Bagherian G (2007). Prediction of cytotoxicity data (CC50) of anti-HIV 5-pheny-l-phenylamino-1H-imidazole derivatives by artificial neural network trained with Levenberg–Marquardt algorithm. J Mol Graph Model.

[3] Asadpour-Zeynali K, Soheili-Azad P (2010). Simultaneous polarographic determination of isoniazid and rifampicin by differential pulse polarography method and support vector regression. Electrochim Acta.

[4] Bolchi C, Pallavicini M, Rusconi C, Diomede L, Ferri N, Corsini A (2007). Peptidomimetic inhibitors of farnesyltransferase with high in vitro activity and significant cellular potency. Bioorg Med Chem Lett.

[5] Cheng Z, Zhang Y, Fu W (2010). QSAR study of carboxylic acid derivatives as HIV-1 Integrase inhibitors. Eur J Med Chem.

[6] Darnag R, Mostapha Mazouz E, Schmitzer A, Villemin D, Jarid A, Cherqaoui D (2010). Support vector machines: development of QSAR models for predicting anti-HIV-1 activity of TIBO derivatives. Eur J Med Chem.

[7] Dastmalchi S, Hamzeh-Mivehroud M, Ghafourian T, Hamzeiy H (2008). Molecular modeling of histamine H3 receptor and QSAR studies on arylbenzofuran derived H3 antagonists. J Mol Graph Model.

[8] Dearden J, Cronin M, Kaiser K (2009). How not to develop a quantitative structure–activity or structure–property relationship (QSAR/QSPR). SAR QSAR Environ Res.

[9] Eastman RT, White J, Hucke O, Yokoyama K, Verlinde CL, Hast MA (2007). Resistance mutations at the lipid substrate binding site of Plasmodium falciparum protein farnesyltransferase. Mol Biochem Parasitol.

[10] Equbal T, Silakari O, Ravikumar M (2008). Exploring three-dimensional quantitative structural activity relationship (3D-QSAR) analysis of SCH 66336 (Sarasar) analogues of farnesyltransferase inhibitors. Eur J Med Chem.

[11] Freitas HF, Castilho MS (2008). 2D QSAR studies of farnesyltransferase inhibitors againts Plasmodium falciparum.

[12] Gaurav A, Gautam V, Singh R (2011). Exploring the structure activity relationships of imidazole containing tetrahydrobenzodiazepines as farnesyltransferase inhibitors: A QSAR study. Lett Drug Des Discov.

[13] Ghasemi S, Davaran S, Sharifi S, Asgari D, Abdollahi A, Mojarrad JS (2013). Comparison of cytotoxic activity of L778123 as a farnesyltranferase inhibitor and doxorubicin against A549 and HT-29 cell lines. Adv Pharm Bull.

[14] Ghasemi S, Sharifi S, Davaran S, Danafar H, Asgari D, Mojarrad JS (2013). Synthesis and cytotoxicity evaluation of some novel 1-(3-Chlorophenyl) piperazin-2-one derivatives bearing imidazole bioisosteres. Aust J Chem.

[15] Gilleron P, Wlodarczyk N, Houssin R, Farce A, Laconde G, Goossens J-F (2007). Design, synthesis and biological evaluation of substituted dioxodibenzothiazepines and dibenzocycloheptanes as farnesyltransferase inhibitors. Bioorg Med Chem Lett.

[16] González MP, Caballero J, Tundidor-Camba A, Helguera AM, Fernández M (2006). Modeling of farnesyltransferase inhibition by some thiol and non-thiol peptidomimetic inhibitors using genetic neural networks and RDF approaches. Biorg Med Chem.

[17] Gupta MK, Prabhakar YS (2008). QSAR study on tetrahydroquinoline analogues as plasmodium protein farnesyltransferase inhibitors: A comparison of rationales of malarial and mammalian enzyme inhibitory activities for selectivity. Eur J Med Chem.

[18] Habibi-Yangjeh A (2009). QSAR study of the 5-HT1A receptor affinities of arylpiperazines using a genetic algorithm–artificial neural network model. Monatsh Chem.

[19] Jain SV, Ghate M, Bhadoriya KS, Bari SB, Chaudhari A, Borse JS (2012). 2D, 3D-QSAR and docking studies of 1, 2, 3-thiadiazole thioacetanilides analogues as potent HIV-1 non-nucleoside reverse transcriptase inhibitors. Org Med Chem Lett.

[20] Jalali-Heravi M, Asadollahi-Baboli M, Shahbazikhah P (2008). QSAR study of heparanase inhibitors activity using artificial neural networks and Levenberg–Marquardt algorithm. Eur J Med Chem.

[21] Katritzky AR, Kuanar M, Slavov S, Hall CD, Karelson M, Kahn I (2010). Quantitative correlation of physical and chemical properties with chemical structure: Utility for prediction. Chem Rev.

[22] Leardi R, Seasholtz MB, Pell RJ (2002). Variable selection for multivariate calibration using a genetic algorithm: prediction of additive concentrations in polymer films from Fourier transform-infrared spectral data. Anal Chim Acta.

[23] Louis B, Agrawal VK, Khadikar PV (2010). Prediction of intrinsic solubility of generic drugs using MLR, ANN and SVM analyses. Eur J Med Chem.

[24] Lu A, Zhang J, Yin X, Luo X, Jiang H (2007). Farnesyltransferase pharmacophore model derived from diverse classes of inhibitors. Bioorg Med Chem Lett.

[25] Ohkanda J, Lockman JW, Yokoyama K, Gelb MH, Croft SL, Kendrick H (2001). Peptidomimetic inhibitors of protein farnesyltransferase show potent antimalarial activity. Bioorg Med Chem Lett.

[26] Olepu S, Suryadevara PK, Rivas K, Yokoyama K, Verlinde CL, Chakrabarti D (2008). 2-Oxo-tetrahydro-1,8-naphthyridines as selective inhibitors of malarial protein farnesyltransferase and as anti-malarials. Bioorg Med Chem Lett.

[27] Puntambekar DS, Giridhar R, Yadav MR (2008). Insights into the structural requirements of farnesyltransferase inhibitors as potential anti-tumor agents based on 3D-QSAR CoMFA and CoMSIA models. Eur J Med Chem.

[28] Shahlaei M, Fassihi A, Saghaie L (2010). Application of PC-ANN and PC-LS-SVM in QSAR of CCR1 antagonist compounds: a comparative study. Eur J Med Chem.

[29] Shayanfar A, Ghasemi S, Soltani S, Asadpour-Zeynali K, Doerksen RJ, Jouyban A (2013). Quantitative structure-activity relationships of imidazole-containing farnesyltransferase inhibitors using different chemometric methods. Med Chem.

[30] Soltani S, Abolhasani H, Zarghi A, Jouyban A (2010). QSAR analysis of diaryl COX-2 inhibitors: comparison of feature selection and train-test data selection methods. Eur J Med Chem.

[31] Tanaka R, Rubio A, Harn NK, Gernert D, Grese TA, Eishima J (2007). Design and synthesis of piperidine farnesyltransferase inhibitors with reduced glucuronidation potential. Biorg Med Chem.

[32] Todeschini R, Consonni V (2008). Handbook of molecular descriptors.

[33] van de Waterbeemd H, Testa B (2008). Drug bioavailability: estimation of solubility, permeability, absorption and bioavailability.

[34] Vapnik V (2000). The nature of statistical learning theory.

[35] Xie A, Sivaprakasam P, Doerksen RJ (2006). 3D-QSAR analysis of antimalarial farnesyltransferase inhibitors based on a 2,5-diaminobenzophenone scaffold. Biorg Med Chem.

[36] Yee LC, Wei YC, Dehmer M, Varmuza K, Bonchev D (2012). Current modeling methods used in QSAR/QSPR. Statistical modelling of molecular descriptors in QSAR/QSPR.

